# Alcohol consumption may not be a risk factor for sarcopenia in the older adults

**DOI:** 10.3389/ebm.2025.10520

**Published:** 2025-05-29

**Authors:** En-Hui Mao, Yun-Ling Bu, Qiao-Ling Liu, Jin-Shui Xu, Xiang Lu, Xi-Lan Yang, Wei Gao, Zheng-Kai Shen

**Affiliations:** ^1^ Department of Endocrinology, Danyang Hospital of Traditional Medicine, Danyang, China; ^2^ Department of General Practice, The Fourth Affiliated Hospital of Nanjing Medical University, Nanjing, China; ^3^ Department of Geriatrics, The First People’s Hospital of Lianyungang, Lianyungang, China; ^4^ Jiangsu Province Center for Disease Control and Prevention, Nanjing, China; ^5^ Department of Geriatrics, Sir Run Run Hospital, Nanjing Medical University, Nanjing, China; ^6^ Department of Geriatrics, Zhongda Hospital, School of Medicine, Southeast University, Nanjing, China

**Keywords:** alcohol, sarcopenia, older adults, community-dwelling, odds ratio

## Abstract

The relationship between drinking and sarcopenia remains controversial. The aim of the present study was to investigate the association of alcohol drinking with sarcopenia in the older adults. A prospective study with 5244 Chinese community-dwelling older adults aged ≥65 years was performed. Sarcopenia was assessed by appendicular skeletal muscle mass index, grip strength, and gait speed. A quantitative questionnaire was used to obtain the information of alcohol drinking. After 4-year follow-up, our study showed that drinkers had lower incidence of sarcopenia than those non-drinkers (19.4% vs. 30.4%, *P* < 0.001 in males and 9.5% vs. 20.4%, *P* = 0.004 in females, respectively). Moreover, male drinkers had higher levels of muscle mass [median (IQR): 7.3 (6.7–7.9) kg/m^2^ vs. 7.1 (6.5–7.7) kg/m^2^, *P* < 0.001] grip strength [median (IQR): 31.1 (26.5–35.0) kg vs. 29.6 (24.8–38.8) kg, *P* < 0.001], and gait speed [median (IQR): 1.08 (0.98–1.17) m/s vs. 1.05 (0.94–1.15) m/s, *P* < 0.001] than those non-drinkers, while female drinkers had higher gait speed [median (IQR): 1.02 (0.94–1.11) m/s vs. 0.99 (0.89–1.09) m/s, *P* = 0.031] than those non-drinkers. Multivariate logistic regression showed that in older adults younger than 85 years, both interim drinking (RR = 0.60; 95%CI = 0.39–0.93; *P* = 0.021 for males; RR = 0.36; 95%CI = 0.13–0.90; *P* = 0.035 for females) and daily drinking (RR = 0.78; 95%CI = 0.61–0.99; *P* = 0.045 for males; RR = 0.34; 95%CI = 0.12–0.96; *P* = 0.041 for females) were correlated with decreased risk of sarcopenia even after adjustment for confounding factors. However, our dose-response analysis did not show any significant relationship between daily alcohol intake and the risk of sarcopenia as well as the components of sarcopenia. In conclusion, our results indicated that alcohol drinking may not be a risk factor for sarcopenia in the older adults. Further research will help to understand the underlying mechanism of the observed causal relationship.

## Impact statement

Although the possible role of alcohol consumption in sarcopenia has attracted increasing attention, the results of current studies remain controversial. Our prospective study provides a novel information that alcohol consumption may not be a risk factor for sarcopenia in a community-dwelling population of Chinese older adults. Specifically, alcohol consumption might have a potential protective effect against the risk of sarcopenia in older adults younger than 85 years old and those who were not underweight (BMI≥18.5 kg/m^2^). However, heavy drinking has significant social burden in addition to human health and therefore is not recommended. Advices regarding the health effects of alcohol drinking on sarcopenia need to be further individualized according to the specific medical status.

## Introduction

Sarcopenia has been identified as a novel geriatric disorder characterized by loss of muscle mass and muscle strength compromising with age [1]. The prevalence of sarcopenia in the older adult ranges from 6% to 12% worldwide [2], and 10.6%–38.8% in China [3–6]. Emerging evidence have linked sarcopenia with a variety of adverse outcomes including frailty, falls, fracture, morbidity and mortality, leading sarcopenia becomes a heavy burden and hotspot in the society of geriatrics [7].

Lifestyle is closely related to sarcopenia, among which drinking is one of the most modifiable behaviors [8]. Drinking is a traditional cultural behavior in China, with the drinking rate of 36.5% and 8.1% in aged male and female, respectively [9]. Although the possible role of alcohol consumption in sarcopenia has attracted increasing attention, the results of current studies remain controversial. Three studies based on Asian population suggested that alcohol drinking might be risk factor for sarcopenia [10–12], whereas another study did not found any correlation between alcohol consumption and sarcopenia [13]. Intriguingly, meta-analysis even indicated that alcohol intake might have protective effect on sarcopenia, especially in males [14, 15], while another recently published meta-analysis reported negative result [16]. The inconsistent results may attribute to the difference of diagnostic criteria for sarcopenia as well as the lack of the specificity of drinking information, such as drinking frequency and consumed alcohol volume. Therefore, the present study aimed to investigate the association of alcohol consumption with sarcopenia in a larger sample size with Chinese community-dwelling older adults.

## Materials and methods

### Study participants

This prospective study was based on participants from the National Basic Public Health Project in Yuetang Community Medical Center in Yangzhou, Jiangsu Province, China in 2020. Participants completed the general information questionnaire (including alcohol intake), physical examinations (including the collection of blood samples), and anthropometry information. A total of 5976 older adults aged ≥65 years were recruited. The exclusion criteria included: (1) unable to accomplish the specified actions (n = 173); (2) had a history of malignant tumors, dementia, mental disorders, severe cardiopulmonary dysfunction (New York Heart Association class III-IV) (n = 537); (3) With missing drinking information (n = 22). Finally, 5244 older adults were included in the follow-up study. This study was performed in accordance with the principles stated in the Declaration of Helsinki [17] and approved by the Ethics Committee of Sir Run Run Hospital, Nanjing Medical University (approval No. 2019-SR-S041). Written informed consent was obtained from each participant.

### Assessment of alcohol consumption

Participants were classified as non-drinkers, interim drinkers (<7 days/week), and daily drinkers, as previously described [18]. For the daily drinkers, we further collected information about the type of alcohol (hard liquor, wine, beer) as well as the amount of intake, which was calculated in grams per day by multiplying the average frequency (times per day) by the amount of each beverage and its corresponding pure ethanol content (5 g ethanol for every 100 g of beer, 12 g ethanol for every 100 g of wine, and 40 g for every 100 g of hard liquor) [12].

### Assessment of sarcopenia

The status of sarcopenia was assessed every year. Muscle mass was measured by bioelectrical impedance analysis (BIA) (Inbody S10; Inbody Korea Ltd., Korea). The height-adjusted appendicular skeletal muscle mass index (ASMI) was calculated as ASM (the sum of skeletal muscle in the arms and legs) divided by height squared in meters (ASM/height [2]). Low muscle mass was defined as an ASMI <7.0 kg/m^2^ in men and <5.7 kg/m^2^ in women [8]. Muscle strength was represented by grip strength measured using a dynamometer (CAMRY EH101, China). Low muscle strength was defined as handgrip strength <28 kg in men and <18 kg in women [8]. Gait speed on a 6-m test <1 m/s was defined as declined physical performance [8].

### Statistical analysis

Kolmogorov-Smirnov test was applied to test the normality of continuous variable. Non-normal data were represented as median and interquartile range (IQR), and compared by Mann-Whitney *U* test. Qualitative variables were represented as frequencies and compared by Pearson *χ2* test. Logistic regression analyses were performed to identified the variables associated with the risk of sarcopenia. Odds ratios (ORs) and 95% confidence intervals (CIs) were calculated. Dose-response analysis was performed by the method described by Greenland and Orsini [19, 20]. The ORs that were the most confounding factors were adjusted and their 95% CIs were used to estimate log ORs and their standard errors (SEs). Linear regression was used to explore the dose-response relationship of every 1g increase in daily alcohol intake with the aspects of sarcopenia. Restricted cubic splines (four knots at fixed percentiles of 5%, 35%, 65%, and 95% of the distribution) were applied to evaluate potential nonlinear dose-response relationship between alcohol consumption and sarcopenia. All statistical analyses were performed by using SPSS 28.0 (IBM SPSS, Inc., USA). All analyses were two-sided and *P* < 0.05 was considered as statistical significance.

## Results

### Baseline characteristics

Among 5244 enrolled subjects, there were 1,050 older adults drank alcohol (20.0%) with 35.9% in males and 4.4% in females. As shown in Table 1, the male drinkers had higher levels of aspartate transaminase (AST)/alanine transaminase (ALT) ratio and total bilirubin (TBIL), whereas the female drinkers had higher levels of hemoglobin (HGB) at baseline (*P* < 0.05). By contrast, there were no significant differences in other clinical parameters as well as the ASMI, grip strength, and gait speed between drinks and non-drinkers at baseline (*P* > 0.05).

**TABLE 1:** Characteristics according to alcohol consumption at baseline.

Variables	Men				Women			
	Total (n = 2,603)	Non-drinker (n = 1,669)	Drinker (n = 934)	*P*	Total (n = 2,641)	Non-drinker (n = 2,525)	Drinker (n = 116)	*P*
Age, y	72 (69–76)	73 (69–78)	73 (68–77)	0.861	73 (69–77)	73 (69–77)	72 (69–76)	0.363
BMI, kg/m^2^	23.5 ± 3.3	23.2 (20.9–25.6)	23.3 (20.5–25.6)	0.763	24.7 (22.4–27.2)	24.7 (22.4–27.3)	24.7 (22.5–26.7)	0.637
Waist-to-hip ratio	0.90 (0.87–0.94)	0.90 (0.86–0.94)	0.90 (0.87–0.93)	0.882	0.90 (0.86–0.94)	0.90 (0.86–0.94)	0.90 (0.87–0.93)	0.630
HGB, g/L	137 (128–144)	137 (126–142)	138 (127–144)	0.221	134 (127–139)	133 (127–139)	135 (129–142)	0.013
WBC, 10^9^/L	5.4 (4.6–6.4)	5.4 (4.6–6.5)	5.4 (4.6–6.4)	0.486	5.3 (4.5–6.3)	5.4 (4.5–6.3)	5.2 (4.6–6.0)	0.652
PLT, 10^9^/L	139 (110–171)	139 (109–170)	140 (110–171)	0.164	146 (116–181)	146 (115–181)	146 (116–182)	0.560
FBG, mmol/L	5.4 (5.1–5.9)	5.4 (5.1–5.9)	5.4 (5.1–5.9)	0.558	5.4 (5.1–6.0)	5.4 (5.1–6.0)	5.5 (5.2–5.9)	0.762
ALB, g/L	37.2 (35.6–43.2)	37.2 (35.3–43.9)	37.3 (35.4–43.5)	0.745	36.5 (34.2–41.2)	36.2 (33.8–43.1)	36.3 (33.4–42.8)	0.152
AST/ALT ratio	1.48 (1.15–1.92)	1.44 (1.13–1.86)	1.57 (1.21–2.00)	<0.001	1.53 (1.20–1.96)	1.52 (1.20–1.96)	1.56 (1.25–1.96)	0.479
TBIL, μmol/L	13.5 (10.6–17.7)	13.2 (10.4–17.2)	14.5 (11.0–19.0)	<0.001	11.8 (9.3–15.1)	11.8 (9.3–15.1)	11.8 (9.2–15.0)	0.780
Cr, μmol/L	64.1 (53.1–75.1)	64.2 (53.5–76.2)	64.1 (52.3–75.6)	0.110	62.5 (53.9–71.6)	62.6 (54.0–71.8)	62.1 (53.8–70.7)	0.413
BUN, mmol/L	5.3 (4.4–6.6)	5.3 (4.5–6.6)	5.4 (4.4–6.7)	0.683	5.3 (4.4–6.4)	5.3 (4.4–6.4)	5.5 (4.5–6.5)	0.184
TC, mmol/L	4.6 (4.1–5.2)	4.6 (4.1–5.1)	4.6 (4.2–5.0)	0.416	4.5 (4.1–5.1)	4.5 (4.0–5.1)	5.6 (4.1–5.3)	0.109
TG, mmol/L	1.1 (0.9–1.6)	1.1 (0.9–1.5)	1.1 (0.8–1.6)	0.775	1.2 (1.1–1.4)	1.2 (1.1–1.5)	1.2 (1.0–1.4)	0.813
LDL-C, mmol/L	1.93 (1.55–2.32)	1.93 (1.59–2.33)	1.92 (1.58–2.32)	0.245	2.18 (1.78–2.58)	2.17 (1.78–2.58)	2.25 (1.82–2.63)	0.304
ASMI, kg/m^2^	7.7 (7.1–8.8)	7.6 (7.1–8.9)	7.7 (7.2–8.9)	0.823	6.1 (5.6–6.7)	6.2 (5.6–6.7)	6.1 (5.7–6.6)	0.954
Grip strength, kg	34.2 (32.3–38.3)	33.6 (31.8–39.8)	34.1 (32.5–40.0)	0.572	20.9 (18.0–23.8)	20.8 (18.0–23.8)	21.4 (19.2–23.5)	0.181
Gait speed, m/s	1.36 (1.06–1.56)	1.35 (1.04–1.65)	1.38 (1.03–1.77)	0.221	1.19 (1.09–1.29)	1.19 (1.09–1.30)	1.18 (1.04–1.30)	0.131

BMI, body mass index; ALB, albumin; ASMI, appendicular muscle mass index; HGB, hemoglobin; WBC, white blood cell; PLT, platelet; FBG, fasting blood glucose; AST, aspartate transaminase; ALT alanine transaminase; TBIL, total bilirubin; Cr, creatinine; BUN, blood urea nitrogen; TC, total cholesterol; TG, triglyceride; LDL-C, low-density lipoprotein cholesterol; HDL-C, high-density lipoprotein cholesterol.

### Association of alcohol consumption with sarcopenia

**FIGURE 1:**
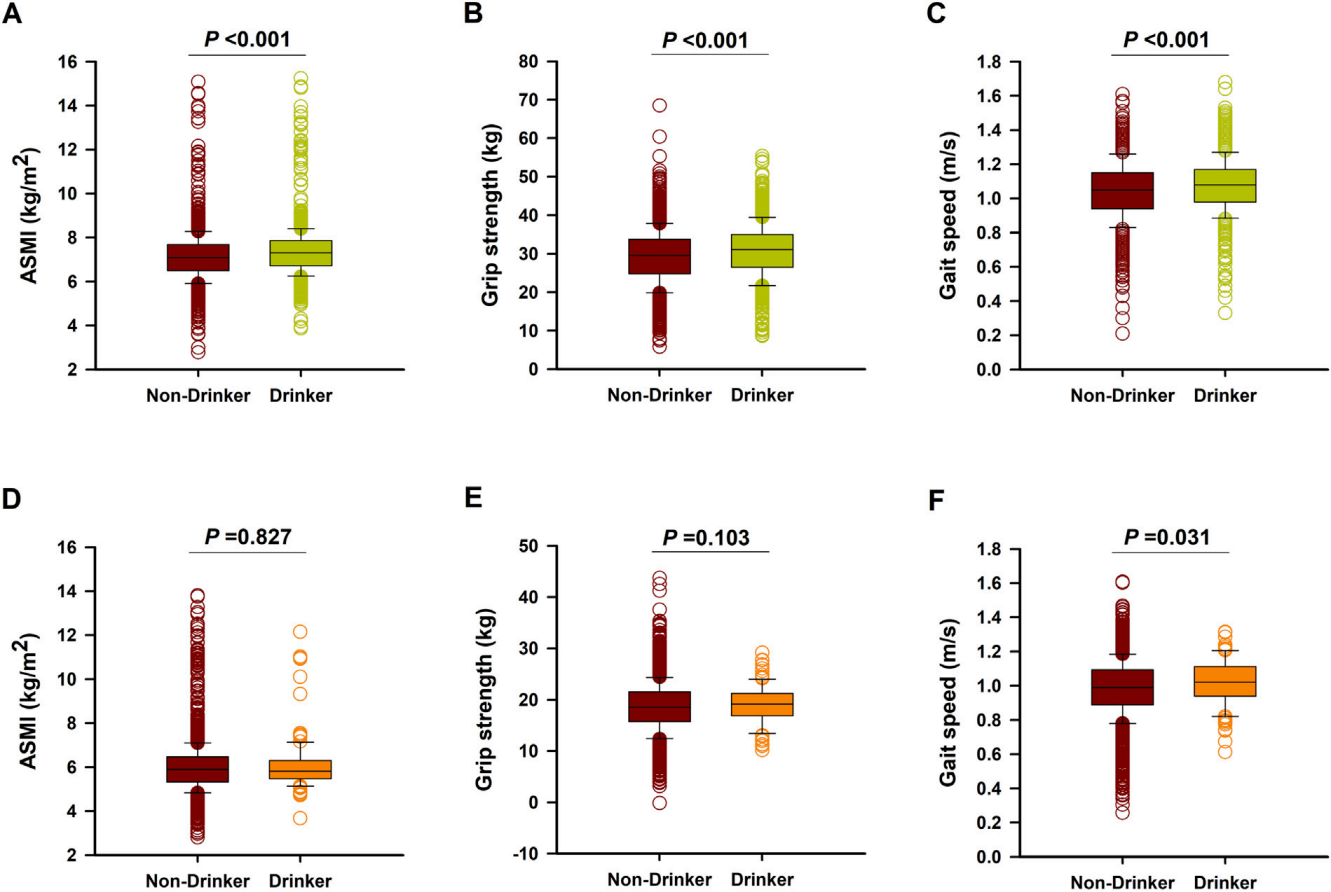
The levels of ASMI, grip strength, and gait speed in male **(A–C)** and Female **(D–F)** non-drinkers and drinkers after 4-years follow-up.

After a follow-up of 4 years, there were 691 aged males had sarcopenia (26.5%), with 184 patients in the drinking group and 507 patients in the non-drinking group. In addition, there were 527 aged females had sarcopenia (20.0%), with 11 patients in the drinking group and 516 patients in the non-drinking group. Interestingly, drinkers had a lower incidence of sarcopenia than those non-drinkers both in males (19.7% vs. 30.4%, *P* < 0.001) and females (9.5% vs. 20.4%, *P* < 0.001). Moreover, male drinkers also had higher levels of muscle mass [median (IQR): 7.3 (6.7–7.9) kg/m^2^ vs. 7.1 (6.5–7.7) kg/m^2^, *P* < 0.001], grip strength [median (IQR): 31.1 (26.5–35.0) kg vs. 29.6 (24.8–38.8) kg, *P* < 0.001], and gait speed [median (IQR): 1.08 (0.98–1.17) m/s vs. 1.05 (0.94–1.15) m/s, *P* < 0.001] than those non-drinkers (Figures 1A–C). By contrast, female drinkers had higher gait speed [median (IQR): 1.02 (0.94–1.11) m/s vs. 0.99 (0.89–1.09) m/s, *P* = 0.031], with no significant difference of muscle mass [median (IQR): 6.0 (5.2–6.4) kg/m^2^ vs. 6.1 (5.2–6.7) kg/m^2^, *P* = 0.827] and grip strength [median (IQR): 19.1 (18.3–21.0) kg vs. 18.6 (17.8–22.3) kg, *P* = 0.103] when compared to those female non-drinkers (Figures 1D–F).

Univariate logistic analysis indicated that the factors correlated with the risk of sarcopenia in males included age, low levels of body mass index (BMI), HGB, white blood counts (WBC), fasting blood glucose (FBG), TBIL, triglyceride (TG), and low-density lipoprotein cholesterol (LDL-C), but higher AST/ALT ratio and high-density lipoprotein cholesterol (HDL-C) (Table 2). Importantly, compared with non-drinkers, older men with interim drinking (RR = 0.56; 95%CI = 0.38–0.81; *P* = 0.003) and daily drinking (RR = 0.56; 95%CI = 0.46–0.69; *P* < 0.001) were less likely to have sarcopenia. For aged females, univariate logistic analysis indicated that the risks factors of sarcopenia included age, low levels of BMI, HGB, FBG, and TG, but higher AST/ALT ratio and HDL-C. Compared with non-drinkers, older women with daily drinking (RR = 0.35; 95%CI = 0.14–0.88; *P* = 0.024) were less likely to suffer from sarcopenia (Table 2). However, these protective effects of drinking on the incidence of sarcopenia were lost after adjustment for potential confounding factors (Table 3). Subgroup analysis suggested that in the older adults with age <85 years old, both interim drinking (RR = 0.60; 95%CI = 0.39–0.93; *P* = 0.021 for males; RR = 0.36; 95%CI = 0.13–0.90; *P* = 0.035 for females) and daily drinking (RR = 0.78; 95%CI = 0.61–0.99; *P* = 0.045 for males; RR = 0.34; 95%CI = 0.12–0.96; *P* = 0.041 for females) were correlated with decreased risk of sarcopenia even after adjustment for confounding factors. Moreover, daily alcohol consumption (RR = 0.75; 95%CI = 0.58–0.95; *P* = 0.019) was also related to decreased risk of sarcopenia in aged males who were not underweight (BMI ≥18.5 kg/m^2^) (Table 3).

**TABLE 2:** Univariate logistic analysis for risk factors of sarcopenia in older adults.

Variables	Men			Women		
	*β*	OR (95%CI)	*P*	*β*	OR (95%CI)	*P*
Age	0.141	1.15 (1.13–1.17)	<0.001	0.143	1.15 (1.13–1.18)	<0.001
BMI	−0.264	0.77 (0.74–0.79)	<0.001	−0.296	0.74 (0.72–0.77)	<0.001
HGB	−0.035	0.97 (0.96–0.97)	<0.001	−0.028	0.97 (0.96–0.98)	<0.001
WBC	−0.090	0.91 (0.86–0.97)	0.003	−0.037	0.96 (0.90–1.03)	0.268
PLT	−0.001	1.00 (1.00–1.00)	0.302	−0.001	1.00 (1.00–1.00)	0.135
FBG	−0.098	0.91 (0.84–0.98)	0.013	−0.077	0.93 (0.86–0.99)	0.041
ALB	0.003	0.98 (0.97–1.03)	0.546	0.008	1.02 (0.98–1.05)	0.411
AST/ALT ratio	0.438	1.55 (1.38–1.74)	<0.001	0.645	1.91 (1.67–2.17)	<0.001
TBIL	−0.019	0.98 (0.97–0.99)	0.008	−0.009	0.99 (0.97–1.01)	0.340
Cr	0.001	1.00 (1.00–1.00)	0.617	0.003	1.00 (1.00–1.00)	0.261
BUN	0.034	1.03 (0.98–1.08)	0.143	0.063	1.07 (1.01–1.13)	0.029
TC	−0.058	0.94 (0.87–1.03)	0.170	0.011	1.01 (0.93–1.10)	0.790
TG	−0.339	0.71 (0.62–0.82)	<0.001	−0.241	0.79 (0.70–0.88)	<0.001
LDL-C	−0.159	0.85 (0.74–0.99)	0.036	−0.095	0.91 (0.78–1.06)	0.220
HDL-C	0.575	1.78 (1.42–2.23)	<0.001	0.868	2.38 (1.82–3.12)	<0.001
Alcohol consumption						
Non-drinker (64.12%)		1 (reference)			1 (reference)	
Interim drinker (7.07%)	−0.584	0.56 (0.38–0.81)	0.003	−0.741	0.48 (0.20–1.12)	0.089
Daily drinker (28.81%)	−0.574	0.56 (0.46–0.69)	<0.001	−1.057	0.35 (0.14–0.88)	0.024

OR, odds ratio; CI, confidence interval; BMI, body mass index; HGB, hemoglobin; WBC, white blood cell; PLT, platelet; FBG, fasting blood glucose; ALB, albumin; AST, aspartate transaminase; ALT alanine transaminase; TBIL, total bilirubin; Cr, creatinine; BUN, blood urea nitrogen; TC, total cholesterol; TG, triglyceride; LDL-C, low-density lipoprotein cholesterol; HDL-C, high-density lipoprotein cholesterol.

**TABLE 3:** Multivariate logistic regression analyses for the association of alcohol consumption with sarcopenia.

Variables	Model 1			Model 2		
	β	OR (95%CI)	*p*	β	OR (95%CI)	*p*
Men						
Overall analyses						
Non-drinker (64.12%)		1 (reference)			1 (reference)	
Interim drinker (7.07%)	−0.283	0.75 (0.50–1.15)	0.187	−0.249	0.78 (0.51–1.19)	0.249
Daily drinker (28.81%)	−0.225	0.80 (0.63–1.01)	0.058	−0.150	0.86 (0.67–1.10)	0.236
Stratification analyses						
Age						
<85 years (n = 2,489)						
Non-drinker (63.08%)		1 (reference)			1 (reference)	
Interim drinker (7.15%)	−0.562	0.57 (0.38–0.87)	0.009	−0.504	0.60 (0.39–0.93)	0.021
Daily drinker (29.77%)	−0.383	0.68 (0.55–0.85)	0.001	−0.247	0.78 (0.61–0.99)	0.045
≥85 years (n = 114)						
Non-drinker (86.84%)		1 (reference)			1 (reference)	
Interim drinker (5.26%)	1.154	3.17 (0.30–33.57)	0.338	1.147	3.15 (0.18–54.19)	0.430
Daily drinker (7.89%)	−0.721	0.49 (0.11–2.20)	0.349	−1.109	0.33 (0.05–2.26)	0.259
BMI						
<18.5 kg/m^2^ (n = 164)						
Non-drinker (81.71%)		1 (reference)			1 (reference)	
Interim drinker (4.27%)	−1.029	0.36 (0.07–1.79)	0.211	−1.240	0.29 (0.05–1.72)	0.172
Daily drinker (14.02%)	−0.553	0.58 (0.23–1.46)	0.245	−0.264	0.77 (0.24–2.44)	0.654
≥18.5 kg/m^2^ (n = 2,439)						
Non-drinker (62.94%)		1 (reference)			1 (reference)	
Interim drinker (7.26%)	−0.221	0.80 (0.53–1.21)	0.296	−0.217	0.81 (0.53–1.23)	0.314
Daily drinker (29.81%)	−0.240	0.79 (0.63–0.99)	0.040	−0.294	0.75 (0.58–0.95)	0.019
Women						
Overall analyses						
Non-drinker (95.61%)		1 (reference)			1 (reference)	
Interim drinker (2.08%)	−0.935	0.39 (0.16–1.01)	0.057	−0.937	0.39 (0.15–1.01)	0.059
Daily drinker (2.31%)	−0.951	0.39 (0.14–1.04)	0.061	−0.886	0.41 (0.15–1.11)	0.080
Stratification analyses						
Age						
<85 years (n = 2,511)						
Non-drinker (95.58%)		1 (reference)			1 (reference)	
Interim drinker (2.07%)	−1.052	0.35 (0.12–0.99)	0.048	−1.022	0.36 (0.13–0.90)	0.035
Daily drinker (2.35%)	−1.135	0.32 (0.11–0.91)	0.033	−1.089	0.34 (0.12–0.96)	0.041
≥85 years (n = 130)						
Non-drinker (96.15%)		1 (reference)			1 (reference)	
Interim drinker (2.31%)	0.385	1.47 (0.13–16.93)	0.758	0.248	1.28 (0.09–18.10)	0.854
Daily drinker (1.54%)	−0.616	0.54 (0.03–9.00)	0.668	−0.443	0.64 (0.03–14.11)	0.779
BMI						
<18.5 kg/m^2^ (n = 111)						
Never drinker (99.10%)	-	-	-	-	-	-
Interim drinker (0.90%)	-	-	-	-	-	-
Daily drinker (0.00%)	-	-	-	-	-	-
≥18.5 kg/m^2^ (2,530)						
Never drinker (95.45%)		1 (reference)			1 (reference)	
Interim drinker (2.13%)	−0.701	0.50 (0.21–1.20)	0.120	−0.816	0.44 (0.18–1.09)	0.075
Daily drinker (2.41%)	−0.841	0.43 (0.17–1.12)	0.085	−0.855	0.43 (0.16–1.11)	0.080

OR, odds ratio; CI, confidence interval; BMI, body mass index.

Model 1: Adjusted for age (for overall analyses and BMI stratification analyses) or BMI (for overall analyses and age stratification analyses).

Model 2: Adjusted for age (for overall analyses and BMI stratification analyses) or BMI (for overall analyses and age stratification analyses), HGB, WBC, PLT, FBG, AST/ALT ratio, TBIL, Cr, BUN, TC, TG, LDL-C, and HDL-C.

### Dose-response relationship between daily alcohol consumption and sarcopenia

**FIGURE 2:**
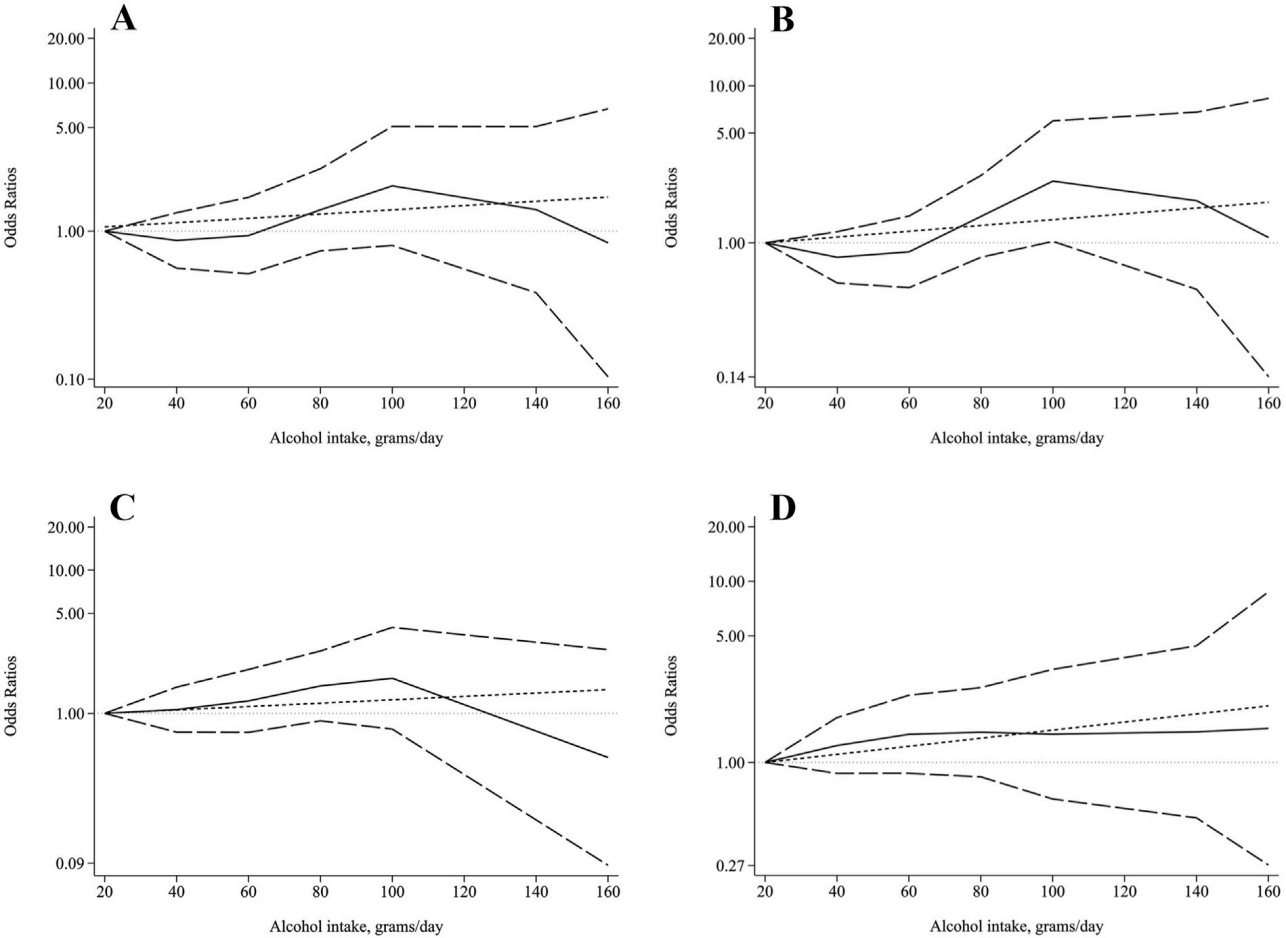
Dose-response relationship between alcohol intake and **(A)** sarcopenia, **(B)** low AMSI, **(C)** low grip strength, and **(D)** low gait speed. The solid line and the long dash line represent the ORs and its 95%CI. Short dash line represents the linear relationship. Linear and spline models were adjusted for age, BMI, HGB, WBC, PLT, FBG, AST/ALT ratio, TBIL, Cr, BUN, TC, TG, LDL-C, and HDL-C.

We further analyzed the dose-response relationship between daily alcohol consumption and sarcopenia. Considering the small number of daily drinkers (n = 61) in the females, we only performed dose-response analysis in the male daily drinkers (n = 750). As shown in Figure 2, restricted cubic splines model indicated no nonlinear dose-response relationship between daily alcohol intake and risk of sarcopenia (*χ2* = 1.76, *P* = 0.414), low muscle mass (*χ2* = 3.68, *P* = 0.159), low grip strength (*χ2* = 2.38, *P* = 0.304), and low gait speed (*χ2* = 0.55, *P* = 0.760). Unfortunately, we did not find linear dose-response relationship between daily alcohol intake and the risk of sarcopenia (*P* = 0.423), as well as low muscle mass (*P* = 0.297), low grip strength (*P* = 0.460), and low gait speed (*P* = 0.156).

## Discussion

Along with the population aging, sarcopenia has been getting more and more attention worldwide. Although various types of lifestyles have been linked to the etiology of sarcopenia [21]; however, the exact correlation between alcohol beverage consumption and sarcopenia remains controversial. Interestingly, we here showed that alcohol drinking might have a potential beneficial effect on the risk of sarcopenia in a community-dwelling population in Yangzhou city of older adults, especially in those younger than 85 years old. Further studies will be needed to identified the appropriate amount of alcohol consumption for the prevention of sarcopenia.

Alcohol consumption is growing globally and accounts for about 5.1% of the global burden of human diseases, especially cardiovascular disease and cancer [22–24]. Animal studies have demonstrated that alcohol can inhibit the synthesis of protein predominately in type II muscle fibers which in turn causes muscle atrophy [25–28]. Moreover, ethanol exposure also induces autophagy flux in C2C12 myotubes, which contributes to the pathogenesis of sarcopenia [26]. However, previous retrospective studies showed controversial results on the relationship between alcohol consumption and sarcopenia. Some studies found detrimental effects of alcohol on the risk of sarcopenia [10–12, 29, 30], whereas others reported negative results between two of them [31–34]. Although the different types of alcoholic beverages, the amount of consumption, as well as the different drinking patterns may affect the relationship between drinking and human diseases [35], the observed protective effects of alcohol intake on the risk of sarcopenia in our present study should be interpreted with caution. According to the dietary guidelines for Chinese residents, the recommended amount of alcohol intake should less than 25g/day [36]. Particularly, daily drinking was associated with lower risk of sarcopenia only in older adults younger than 85 years. This age difference in the relationship between alcohol consumption and sarcopenia may be attribute to the decreased alcohol intake along with aging [37]. Moreover, drinking pattern, such as whether the alcohol is taken at mealtimes [38], may also influence the effect of alcohol consumption on sarcopenia. Interestingly, a previous study showed that drinking with meals might be beneficial for decreasing the all-cause mortality [39]. However, we did not find a significant dose-response relationship between alcohol intake and sarcopenia. Considering that the individual dose was obtained by self-report, the limitation in measurement precision could obscure such a relationship. Therefore, further studies focused on different drinking pattern will help us to understand the potential health effects of alcohol on sarcopenia.

There are some potential explanations that may account for the protection effects against sarcopenia by alcohol consumption. Since malnutrition, especially with low BMI (<18.5 kg/cm^2^), has been demonstrated as one of the most important risk factors for sarcopenia [7], the leading possibility might be related to the weight gain in alcohol consumers, especially in those daily drinkers [40]. However, considering that weight gain induced by alcohol consumption primarily due to an increase in fat mass, which often contributes to obesity, further studies are needed to confirm the association of drinking with the detailed amount of body composition. Another potential reason may be related the relatively positive life attitude of people who drink moderate amounts of alcohol [41]. In the present study, older adults with interim drinking also had a lower risk of sarcopenia. These people may have more social intercourse and exercise, both of which are important preventive measures for the management of sarcopenia [7]. Moreover, another study reported that alcohol consumption could also increase the level of HDL-C [42], which is inversely correlated with the risk of sarcopenia [43]. Sphingolipids level is also decreased in drinkers when compared to those non-drinkers [44]. As an important bioactive sphingolipid, previous studies have shown that ceramide can directly activate atypical PKC isoform protein kinase Cζ to induce endoplasmic reticulum stress and mitochondrial dysfunction [45], which play crucial roles in the pathogenesis of sarcopenia [46]. Therefore, moderate alcohol may play a protective role in skeletal muscle by depleting sphingolipids, especially ceramide. Nevertheless, further in‐depth experiments are needed to investigate the explicit mechanism governing the effects of different types and amount of alcohol on the development of sarcopenia.

Several limitations should still be considered in the present study. Firstly, the information of alcohol consumption was self-reported, which may cause social desirability bias and recall error. Future prospective studies with an intervention of different amount of alcohol will help to determine the dose-dependent effects of alcohol consumption on the prevention of sarcopenia. Secondly, almost all the older adults in this study consumed liquor. The effects of different types of alcohol beverages such as wine, beer, sake, yellow rice, *etc.* on the risk of sarcopenia will need to be assessed. Thirdly, we cannot rule out the possibility of other potential confounding dietary factors, such as the nutritional status, physical activity, and socio-economic conditions as well as the consumption of tea, coffee, fruits, vitamins or other antioxidant substances.

## Conclusion

In summary, our findings suggest that alcohol intake may not be a risk factor for the development of sarcopenia in this Yangzhou cohort. Instead, appropriate amount of alcohol consumption might be related to decreased risk of sarcopenia in older adults younger than 85 years. However, heavy drinking has significant social burden in addition to human health and therefore is not recommended. Advices regarding the health effects of alcohol drinking on sarcopenia need to be further individualized according to the specific medical status.

## Data Availability

The raw data supporting the conclusions of this article will be made available by the authors, without undue reservation.
